# Diversity, community structure, and abundance of *nir*S-type denitrifying bacteria on suspended particulate matter in coastal high-altitude aquaculture pond water

**DOI:** 10.1038/s41598-024-56196-x

**Published:** 2024-03-07

**Authors:** Kuang Chunyi, Sun Wei, Wei Mingken, Xia Chunyu, Li Changxiu

**Affiliations:** 1 College of Biological and Food Engineering, Guangdong University of Petrochemical Technology, Maoming, 525000 People’s Republic of China; 2 College of Life and Geographic Sciences, Kashi University, Kashi, 844000 People’s Republic of China

**Keywords:** Suspended particulate matter, Aquaculture ecosystem, Coastal high-altitude aquaculture pond, *Nir*S gene, Denitrifying bacteria, Ecology, Microbiology, Ecology, Environmental sciences

## Abstract

Denitrifying bacteria harboring the *nitrate reductase* S (*nir*S) gene convert active nitrogen into molecular nitrogen, and alleviate eutrophication in aquaculture water. Suspended particulate matter (SPM) is an important component of aquaculture water and a carrier for denitrification. SPM with different particle sizes were collected from a coastal high-altitude aquaculture pond in Maoming City, China. Diversity, community structure, abundance of *nir*S-type denitrifying bacteria on SPM and environmental influencing factors were studied using high-throughput sequencing, fluorescence quantitative PCR, and statistical analysis. *Pseudomonas*, *Halomonas*, and *Wenzhouxiangella* were the dominant genera of *nir*S-type denitrifying bacteria on SPM from the ponds. Network analysis revealed *Pseudomonas* and *Halomonas* as the key genera involved in the interaction of *nir*S-type denitrifying bacteria on SPM in the ponds. qPCR indicated a trend toward greater *nir*S gene abundance in progressively larger SPM. Dissolved oxygen, pH, temperature, and SPM particle size were the main environmental factors influencing changes in the *nir*S-type denitrifying bacterial community on SPM in coastal high-altitude aquaculture pond water. These findings increase our understanding of the microbiology of nitrogen cycle processes in aquaculture ecosystem, and will help optimize aquatic tailwater treatment strategies.

## Introduction

High-altitude aquaculture ponds with higher breeding densities had become one of the main modes of shrimp breeding, to achieve higher shrimp production^1^. High-altitude aquaculture ponds are usually built on higher water lines, with plastic film covering the soil surface. Pollutant discharge depends on a drainage system at the bottom of the pond^2,3^. The accumulation of harmful substances caused by excessive feeding in high-altitude aquaculture ponds leads to deterioration in water quality, the frequent occurrence of diseases in shrimp ponds, and annual economic losses^2–4^. The large accumulation of organic matter in the late stages of high-density shrimp culture leads to uncontrolled water quality^1,5,6^. In aquatic ecosystems, some microorganisms use different forms of nitrogen, including nitrate nitrogen (NO_3_^−^–N), nitrite nitrogen (NO_2_^−^–N), total ammonia nitrogen (NH_4_^+^–N), and total Kjeldahl nitrogen, as nutrients that are necessary for their metabolism, which contributes to the circulation of nitrogen in the water^7^.

In aquaculture ecosystems, environmental microorganisms are important in productivity, nutrient cycling, and water quality^8^. Denitrification is highly dependent on the activity of microorganisms, such as bacteria and Archaea^9^. Denitrification, an indispensable part of the nitrogen cycle, is the reduction of nitrate to nitrite as catalyzed by nitrate reductase (*Nir*) under anaerobic or microaerobic conditions^10,11^. Subsequently, nitrite is reduced to nitric oxide (NO) as catalyzed by nitrite reductase and then reduction to nitrous oxide (N_2_O) as catalyzed by nitrogen oxide reductase^10,11^. Finally, N_2_O is reduced to molecular nitrogen by nitrogen oxide reductase^10,11^. Marine ecosystems remove 45% of the earth 's nitrogen by denitrification in estuarine and coastal, adjacent sea, and shelf sediments^12^. Moreover, denitrification converts active nitrogen in an ecosystem into molecular nitrogen, which is sufficient to alleviate the occurrence of eutrophication in aquatic ecosystems^13^. In-depth study of denitrification mechanisms increased the understanding of the transformation and removal of active nitrogen from ecosystems. Studies on denitrifying bacteria have often focused on key reductase functional genes^14,15^. *Nir* is the key enzyme and its rate of activity is the rate-limiting step in the denitrification process^15^. *Nir* comprises two isozymes with similar functions but different structures: a copper-containing nitrite reductase encoded by *nir*K and a cytochrome cd1 nitrite reductase encoded by *nir*S^16–18^. The phylogenetic signal of *nir*S is consistent with that of the 16S rRNA gene at the family and genus levels^19^. Therefore, *nir*S is a commonly used molecular marker for characterizing the diversity and abundance of denitrifying bacteria in environmental communities^20,21^.

Suspended particulate matter (SPM) comprises both suspended matter and sediment-derived particles^22^. SPM play a critical role in balancing biogeochemical cycles in aquatic ecosystems^22^. SPM provides a carrier for denitrifying bacteria and supports their role in water denitrification^22^. To obtain nutrients, microorganisms change the pH and oxidation–reduction potential inside SPM and create a microenvironment where enzyme activity increases^23^. Research on SPM denitrification has been mostly limited to estuaries and rivers. Relevant studies in the estuary of Hangzhou Bay in China have shown that denitrifying bacteria mostly exist in the form of aggregates on SPM in water, and that the diversity of denitrifying functional genes positively correlates with the concentration of SPM^22^. A study involving the Yellow River showed the rate of denitrification progressively increased as the SPM concentration in the water increased^24^. However, It is still limited for understanding the community structure and diversity characteristics of denitrifying taxa on SPM in high-altitude aquaculture ponds.

The *nir*S-type denitrifying bacteria are widely distributed in nature. Thus, it is important to quantitatively detect their gene abundance and study the characteristics of microbial communities in different environments^25^. Quantitative polymerase chain reaction (qPCR) is useful to analyze the abundance of *nir*S-type functional genes^25,26^. Zhang et al.^27^ used qPCR to quantitatively detect the abundance of *nir*S-type denitrifying bacteria in the aerobic water layers of the Jinpen and Lijiahe drinking water reservoirs. Zhu et al.^22^ used qPCR to quantitatively detect the abundance of *amo*A and *nir* genes, and reported a correlation between gene abundance and SPM. Xiang et al.^28^ used qPCR to detect the abundance of the key denitrification functional genes *nir* and *nos*Z in sediments of the northern South China Sea. High-throughput Illumina sequencing technology is often used to study bacteria in different environments, and the findings have deepened the understanding of the characteristics of various microflora^1,29,30^. Xiang et al.^28^ revealed the ecological distribution and diversity of denitrification functional genes in sediments through Illumina sequencing; the findings emphasized the role of these key functional genes in potential N_2_O emissions from surface sediments in the northern South China Sea. Shahraki et al.^31^ revealed changes in freshwater bacterial community composition using Illumina sequencing.

In this study, we used high-throughput Illumina sequencing and qPCR technology to comprehensively clarify the community structure, diversity characteristics, and gene abundance of *nir*S-type denitrification functional taxa on SPM with different particle sizes in a high-altitude aquaculture pond. The interactions between the *nir*S-type denitrifying bacteria on SPM in the ponds were explored using network analysis. The relationship between key genera (OTU) and environmental factors were investigated using redundancy analysis (RDA). The results of this study lay a theoretical foundation for understanding the nitrogen removal pathways mediated by SPM in a high-altitude aquaculture pond, contributing to the maintenance of the aquaculture water environment and optimizing wastewater treatment strategies.

## Results and discussion

### Environmental parameters in the high-altitude aquaculture ponds

The results of the physical and chemical indices of the four sampling sites (K1-4) in the high-altitude aquaculture ponds were presented in Table 1. DO concentration ranged from 6.18 to 8.48 mg L^−1^. The average chemical oxygen demand (COD) was 4.59 mg L^−1^. Turbidity fluctuated from 11.86 to 54.10 nephelometric turbidity units (NTU). Total suspended solid (TSS) ranged from 7.00 to 96.00 mg L^−1^. The average concentration of NH_4_^+^–N was 0.02 mg L^−1^; the highest concentration was 0.05 mg L^−1^ at the K2 site. The average concentration of NO_2_^−^–N was 0.03 mg L^−1^; the highest concentration was 0.10 mg L^−1^ at the K3 site. The average value of NO_3_^−^–N was 0.05 mg L^−1^; the highest density was 0.07 mg L^−1^ at the K4 site. Total phosphate (TP) ranged from 3.46 to 29.97 mg L^−1^. The trend of change of active phosphate concentration was consistent with that of TP. The highest concentration of active phosphate was 20.27 mg L^−1^ at the K3 site. In addition, the K3 site had a higher COD value (6.12 mg L^−1^) and NO_2_^−^-N (0.10 mg L^−1^), while the NH_4_^+^-N (0.00 mg L^−1^) and NO_3_^−^–N (0.00 mg L^−1^) values were lower. This may be because nitrogen pollutants, mainly NH_4_^+^-N, were mostly oxidized to NO_3_^−^-N in the aerobic water environment. Therefore, the concentration of NO_2_^−^-N was much higher than those of NH_4_^+^–N and NO_3_^−^–N^22^. Environmental factors were significantly different between K1 and K2 (F = 4.850, *P* = 0.040), K2 and K3 (F = 9.537, *P* = 0.006), and K3 and K4 (F = 7.889, *P* = 0.011) (Supplementary Table S2), indicating niche differences in environmental factors among the sampling sites.

**Table 1:** Means (± SD) of environmental parameters of the four sampling sites (K1-4) in the high-altitude aquaculture ponds.

	Unit	K1	K2	K3	K4
DO	mg L^−1^	8.48 ± 0.31	6.88 ± 0.07	7.38 ± 0.17	6.18 ± 0.03
pH	/	8.10 ± 0.30	8.90 ± 0.10	8.20 ± 0.00	8.00 ± 0.10
Temperature	℃	20.90 ± 0.00	20.60 ± 0.00	20.20 ± 0.30	19.70 ± 0.10
COD	mg L^−1^	5.76 ± 0.04	3.08 ± 0.04	6.12 ± 0.04	3.40 ± 0.04
Turbidity	NTU	38.36 ± 0.23	11.86 ± 0.24	54.10 ± 0.16	14.65 ± 0.24
TSS	mg L^−1^	96.00 ± 3.00	7.00 ± 1.00	52.00 ± 1.00	17.00 ± 2.00
NH_4_^+^–N	mg L^−1^	0.01 ± 0.00	0.05 ± 0.00	0.00 ± 0.00	0.01 ± 0.00
NO_2_^−^–N	mg L^−1^	0.01 ± 0.00	0.01 ± 0.00	0.10 ± 0.00	0.01 ± 0.00
NO_3_^−^–N	mg L^−1^	0.06 ± 0.00	0.05 ± 0.00	0.00 ± 0.00	0.08 ± 0.01
TP	mg L^−1^	10.07 ± 0.09	3.46 ± 0.23	29.97 ± 0.93	4.55 ± 0.09
PO_4_^3−^–P	mg L^−1^	4.43 ± 0.19	1.09 ± 0.08	20.27 ± 0.08	1.44 ± 0.12

The K1-4 is the sampling sites. NO_2_^−^–N stands for nitrite, NO_3_^−^–N stands for nitrate, NH_4_^+^–N represents ammonia, PO_4_^3−^–P stands for active phosphate.

### Analysis of high-throughput sequencing results

Samples with fewer than 380 sequences were removed, and 1264 sequences were screened for each sample. The Mothur platform was used for pretreatment and cluster analysis. A total of 36,249 high-quality sequences were obtained, and 169 operational taxonomic units (OTUs) were obtained by cluster analysis. The dilution curves were presented in Supplementary Figure S1. The trends towards flattening indicate that the sequencing depth covered all species in all samples, and species diversity in the samples was detected. The curve of the Shannon index diagram rapidly increased to a certain value and tended to flatten, as shown in Supplementary Figure S2, indicating that the amount of data sequenced in this study was sufficiently large to reflect the vast majority of microbial information in all samples. The rapid decline in the rank-abundance curve showed that the proportion of dominant bacteria in all samples was high and the diversity was low, as shown in Supplementary Figure S3.

### Diversity of *nir*S-type denitrifying bacterial community in the high-altitude aquaculture ponds

The alpha diversity indices were shown in Table 2. Good reliability and credibility were indicated by the > 97.41% coverage of all samples. The highest number of OTUs in the samples was found in K4_5 and K4_0.22 (121). The sample with the highest Chao1 value was K4_5 (131.30). The Chao1 values in samples K1_5, K1_1, K2_1, K3_0.22 were lower than 100. The highest Ace value was 126.23 in sample K4_0.22. The Shannon values were higher than 3.00 in samples K2_1, K2_0.22, K3_5, and K4_0.22, and lower than 3.00 in the rest of the samples. The highest Simpson value was 0.26 in samples K3_0.22 and K4_1. In terms of sampling sites, K4 had the highest community richness and K2 had the highest community diversity. In terms of particle size, the number of OTUs, community richness and community diversity of were higher on the SPM with 0.22 μm, than those with 5 μm and 1 μm, respectively.

**Table 2:** The results of the alpha diversity of the sample in the high-altitude aquaculture ponds.

Sample	Cov.	OTUs	Chao1	Ace	Shan.	Simp.
K1_5	98.10	86	87.74	97.93	2.55	0.13
K1_1	97.77	94	98.44	122.32	2.52	0.16
K1_0.22	97.67	106	108.83	120.83	2.89	0.09
K2_5	97.95	101	111.51	107.20	2.89	0.13
K2_1	99.05	88	91.67	94.29	3.41	0.06
K2_0.22	98.26	110	113.94	110.74	3.46	0.06
K3_5	98.13	97	102.81	108.14	3.02	0.08
K3_1	97.81	101	101.31	112.55	2.36	0.21
K3_0.22	98.16	94	93.32	95.09	2.18	0.26
K4_5	97.41	121	131.30	137.47	2.96	0.10
K4_1	97.65	110	114.90	121.56	2.39	0.26
K4_0.22	97.80	121	123.55	126.23	3.27	0.09

Simp. stands for Simpson index, Chao1 stands for Chao1 estimator, Shan. stands for Shannon index, Cov. stands for Coverage. The K1-4 is the sampling sites. The number 5, 1, and 0.22 is particle sizes of 5, 1, and 0.22 μm on SPM.

### Community composition of *nir*S-type denitrifying bacteria in the high-altitude aquaculture ponds

**Figure 1:**
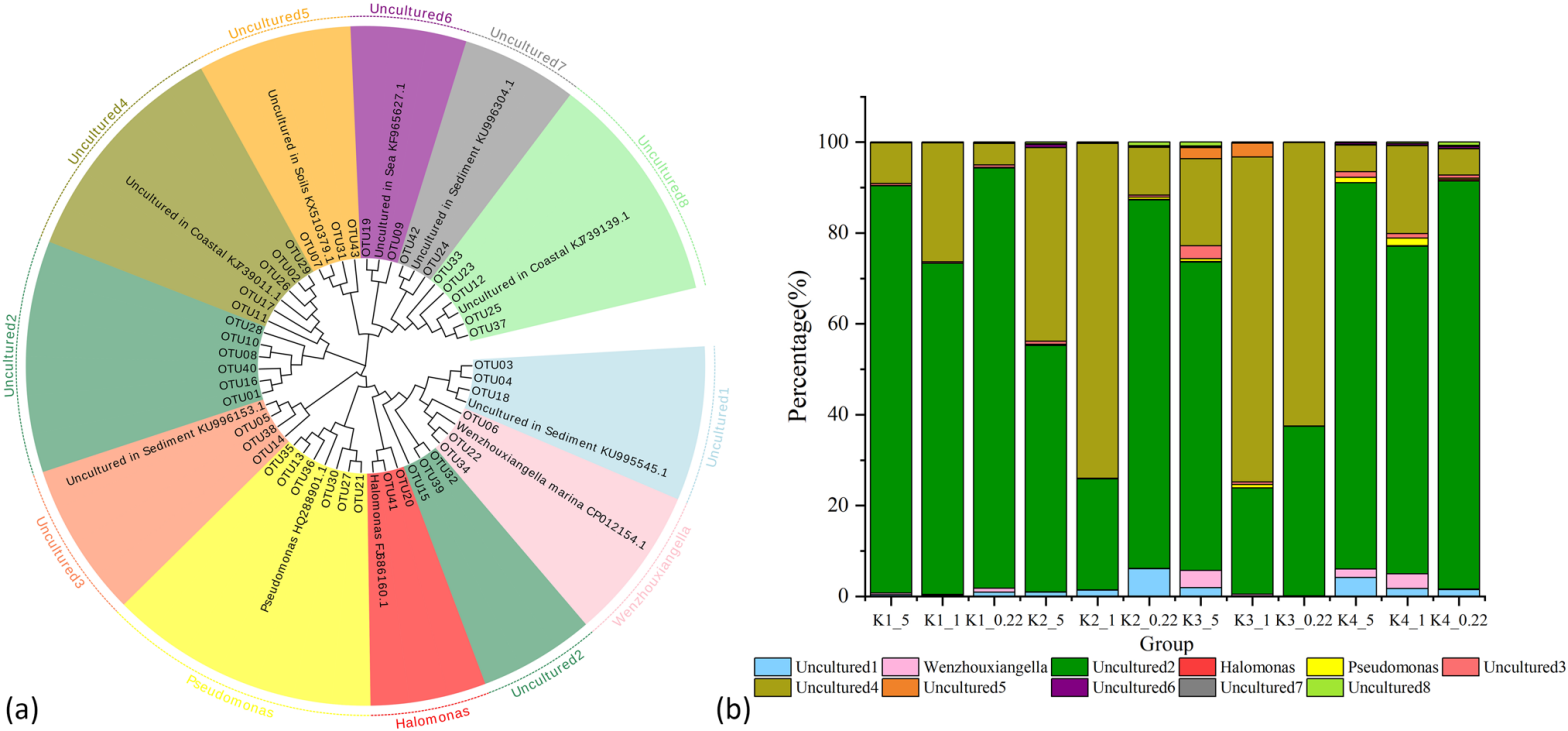
Microbial characteristics of the *nir*S gene on SPM in the high-altitude aquaculture ponds. (**a**) Neighbor-joining phylogenetic tree of dominant OTUs for the *nir*S gene (Top 43 OTUs) and the reference sequences from Genbank. Each color block is a genus classification, and the outer ring is the name of each cluster. (**b**) A cluster division of the neighbor-joining phylogenetic tree. The abscissa in the figure is the sample (The K1-4 is the sampling sites. The number 5, 1, and 0.22 is particle sizes of 5, 1, and 0.22 μm on SPM.). The ordinate is the percentage of each cluster, and different color blocks represent different bacterial classifications.

**Figure 2:**
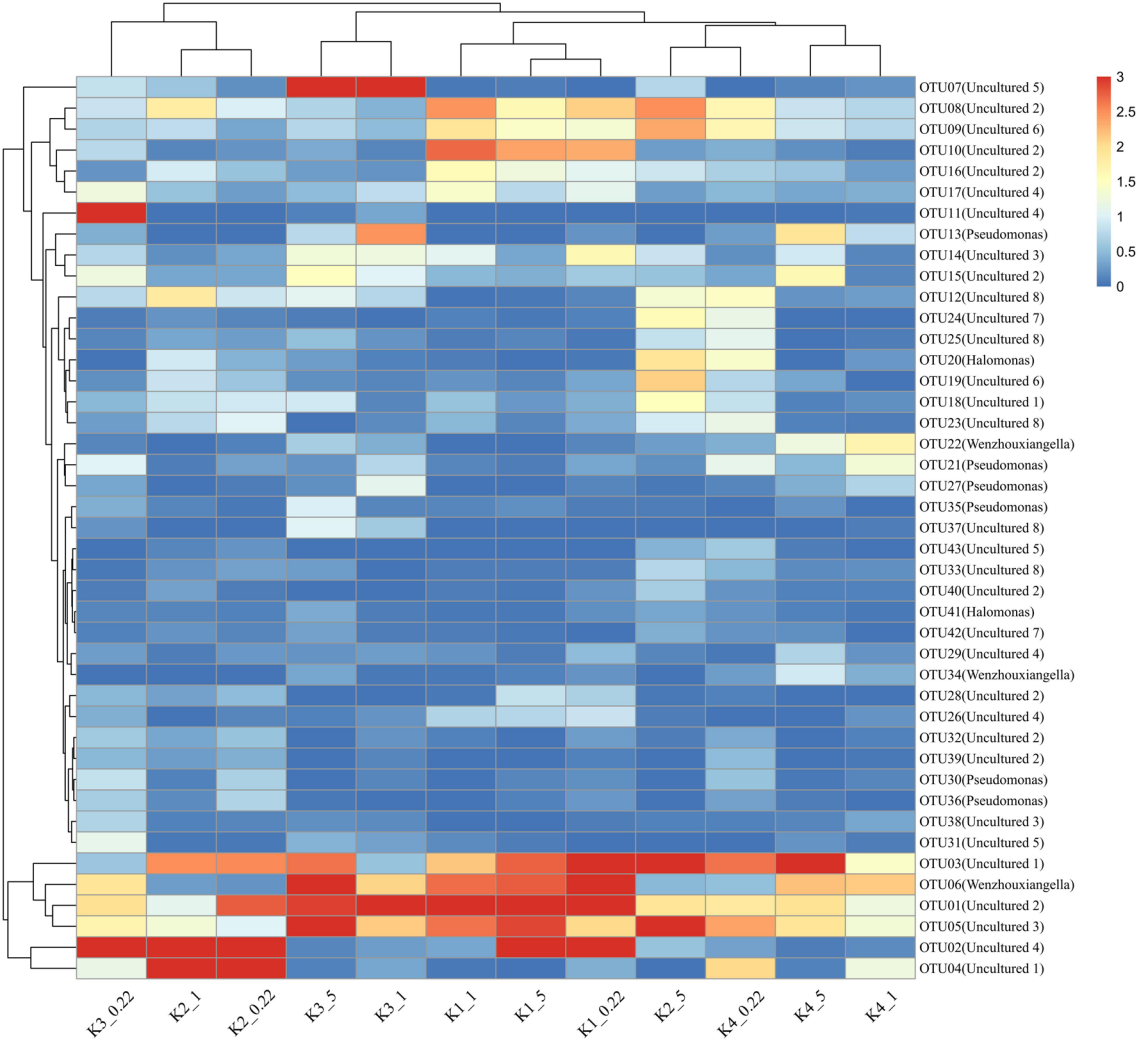
The heat map of dominant OTUs for the *nir*S gene (Top 43 OTUs). The abscissa in the figure represents the sample, and the upper tree diagram represents the sample clustering (The K1-4 is the sampling sites. The number 5, 1, and 0.22 is particle sizes of 5, 1, and 0.22 μm on SPM.). The ordinate is dominant OTUs for the *nir*S gene (Top 43 OTUs), and the left tree diagram represents the clustering of OTU samples. The color square represents the relative abundance of the dominant OTU, and the redder the color, the higher the abundance.

According to *nir*S sequence analysis, 46 OTUs (sequence number > 0.2%) were dominant, accounting for 91.7% of the total sequence number. Based on the OTU clustering results, a phylogenetic tree was constructed using representative sequences of significant OTUs (Fig. 1a). The community structure of *nir*S-type denitrifying bacteria was plotted according to the cluster division of the phylogenetic tree (Fig. 1b). Niche differentiation was observed between the sampling sites. Cluster analysis showed that Proteobacteria were the dominant phylum of *nir*S functional bacteria on the SPM of the coastal high-altitude aquaculture pond water, accounting for 5.02% of the total effective bacterial sequences. *Halomonas*, *Pseudomonas* and *Wenzhouxiangella* of Gamma-Proteobacteria were the dominant genera of *nir*S-type denitrifying bacteria on the SPM. They were distributed in each SPM sample, but their relative abundances varied. The relative abundance of *Wenzhouxiangella* was the highest, accounting for 0.01–3.85% of the total effective bacterial sequence, with an average of 0.93%. *Pseudomonas* accounted for 0.01–1.63% of the total effective bacterial sequences, with an average of 0.44%. *Halomonas* accounted for 0.01–0.29% of the total effective bacterial sequences, with an average of 0.09%. Many uncultured bacteria on the SPM were evident, similar to species from different environments (sediments, estuaries, oceans, and soil). A heat map was drawn according to the clustering results and the relative abundance distribution of the dominant OTUs (Fig. 2). The OTUs were clustered according to the sites. OTU01, 03, 05, and 06 were widespread in all samples. OTU08, 09, 10, 16, 17 were clearly clustered in K1. OTU12, 18, 19, 20, 23, 24, and 25 were clearly clustered in K2 and K4_0.22. OTU11, 13, 14, and 15 obviously clustered at the K3 site.

Proteobacteria was the dominant phylum in the microbial community structure in marine water and sediment environments. The most common *nir*S genotype of denitrifying bacteria in various ecological environments was Proteobacteria^32,33^. Proteobacteria was the dominant bacteria in the water and bed mud of conventional shrimp ponds^1,8,34,35^. In this study, most denitrifying bacteria in the high-altitude aquaculture pond water were unclassified and uncultured, while the rest were mostly Proteobacteria. The high abundance of Proteobacteria in recirculating mariculture system has been described^36^. Therefore, it was conceivable that similar dominant phyla were present in the high-altitude and conventional shrimp ponds. This indicates that Proteobacteria can colonise the surrounding environment under different breeding modes. Proteobacteria was significant for bacterial communication and nitrogen cycling in high-altitude aquaculture ponds. In addition to degrading nutrients, such as amino acids, proteins, and carbohydrates, Proteobacteria was also indispensable role in the denitrification process^4,9^, which could cooperate with Bacteroidetes and Chloroflexi for denitrification in high-altitude aquaculture ponds and other marine recirculating aquaculture systems^36^. Proteobacteria and Bacteroidetes were abundant in marine recirculating aquaculture system biofilters^37,38^ and the sub-Antarctic Southern Ocean^39^. Proteobacteria play indispensable roles in microbial metabolism and geochemical cycles in lanthanide-rich deep environments^40^.

*Halomonas*, *Pseudomonas* and *Wenzhouxiangella* were detected in the samples used in this study. The denitrifying bacteria on the SPM from Hangzhou Bay were mainly *Bradyrhizobium*, *Comamonas*, *Thauera* and others, which showed significant correlations with SPM^22^. Studies have shown that some strains in *Pseudomonas* have good aerobic denitrification ability and can effectively remove nitrates^41^. In the past, denitrification was believed to require strict anoxic conditions for a long time. However, in the early 1980s, aerobic denitrifying bacteria using both oxygen and nitrate as electron acceptors were first reported^42^. Since then, aerobic denitrifying bacteria have been isolated from nutrient-rich systems, such as aquaculture ponds^43,44^. This was because periplasmic-bound nitrate reductase (Nap) present in *Pseudomonas* plays an important role under both aerobic and anaerobic conditions and was essential for nitrate conversion^45,46^. *Halomonas* was mainly composed of marine halophilic aerobic heterotrophic organisms with diverse metabolisms^47^. *Halomonas* can accumulate polyhydroxyalkanoates (PHA) to cope with nutrient depletion conditions and has a specific osmotic adaptation mechanism to prevent molecular damage caused by cell freezing and dehydration^47^. Therefore, *Halomonas* can survive in extremely high-salt environments, such as high-salt lakes^48^, high-salt soils^49^, and deep seas^50^. *Wenzhouxiangella* was first proposed by Wang et al.^51^, using *Wenzhouxiangella marina* KCTC 42284T as the model species. Subsequently, Zhang^52^, Guo^53^, and Han et al.^54^ successively discovered *Wenzhouxiangella* bacteria in waters with high salt concentrations, such as Yuncheng Salt Lake in Shanxi Province, Xiaoshi Island in Weihai, and Xinjiang Salt Lake. Although *Wenzhouxiangella* bacteria were rarely found in aquaculture ponds, it was not surprising that they were found in this coastal high-altitude aquaculture pond, because they can grow under elevated NaCl (optimum 5%) conditions^52^. In addition, *Wenzhouxiangella* contains catalase, aldose dehydrogenase, nitrite reductase, and other genes^52^, indicating that it can use a variety of carbon sources, and also reduce nitrate to nitrite, which plays an important role in the carbon and nitrogen cycle.

**Figure 3:**
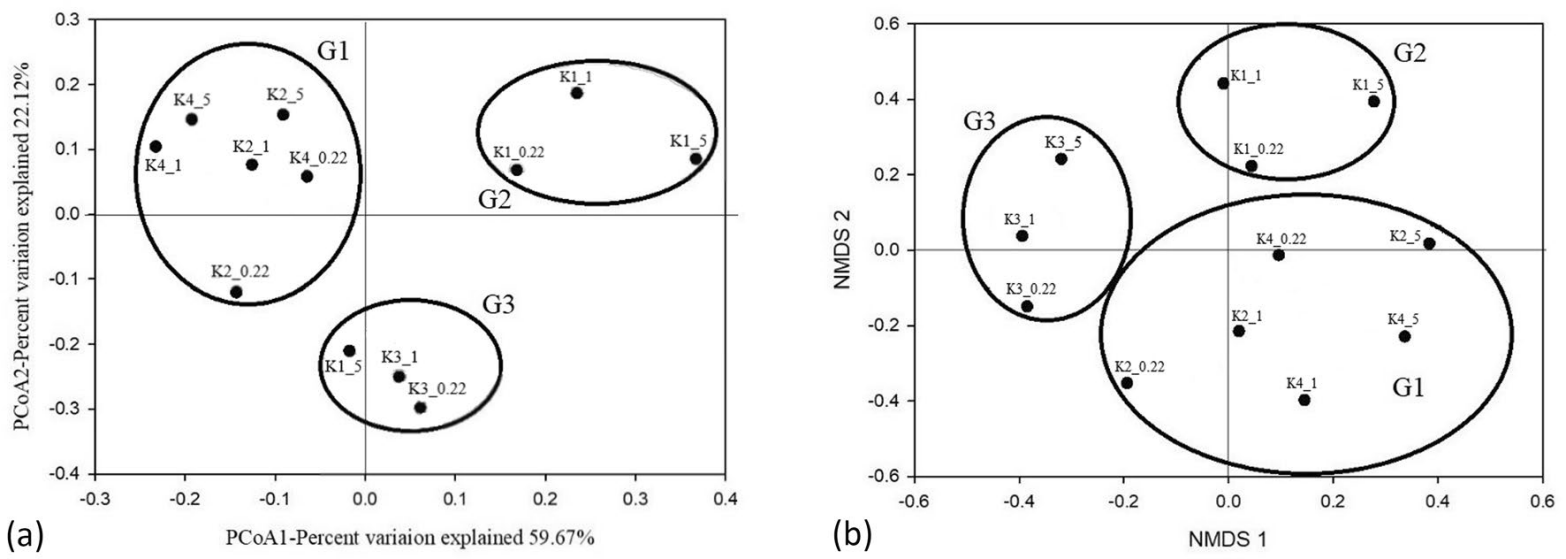
(**a**) The results of PCoA. (**b**) The results of NMDS. The black dots in the figure represent the sample name (The K1-4 is the sampling sites. The number 5, 1, and 0.22 is particle sizes of 5, 1, and 0.22 μm on SPM.). The large circle represents the clustering grouping of each point, and G1-3 represents the different clustering groups.

The results of a principal component analysis (PCoA) were presented in Fig. 3a. The contribution rate of the first and second axes was 59.67% and 22.12%, respectively. Bacterial community construction was similar and clustered together at the K2 and K4 sites. Community construction at the K1 site was significantly different from that at the other sampling sites. The clustering of bacteria was clearly different at K3 with different particle sizes. The non-metric multidimensional scaling (NMDS) results were consistent with those of the PCoA analysis, as shown in Fig. 3b. The bacterial communities formed three groups. The first group (G1) included sites K2 and K4, the second group (G2) included site K1, and the third group (G3) included site K3. Overall clustering (PCoA, NMDS) showed that the bacterial community structure was clustered according to different sampling sites in the high-altitude aquaculture pond water. The clustering of the K2 and K4 sites was due to similar ecological environments, such as Turbidity, TSS, TP, and concentrations of active phosphate.

### Abundance distribution of *nir*S gene in the high-altitude aquaculture ponds

**Figure 4:**
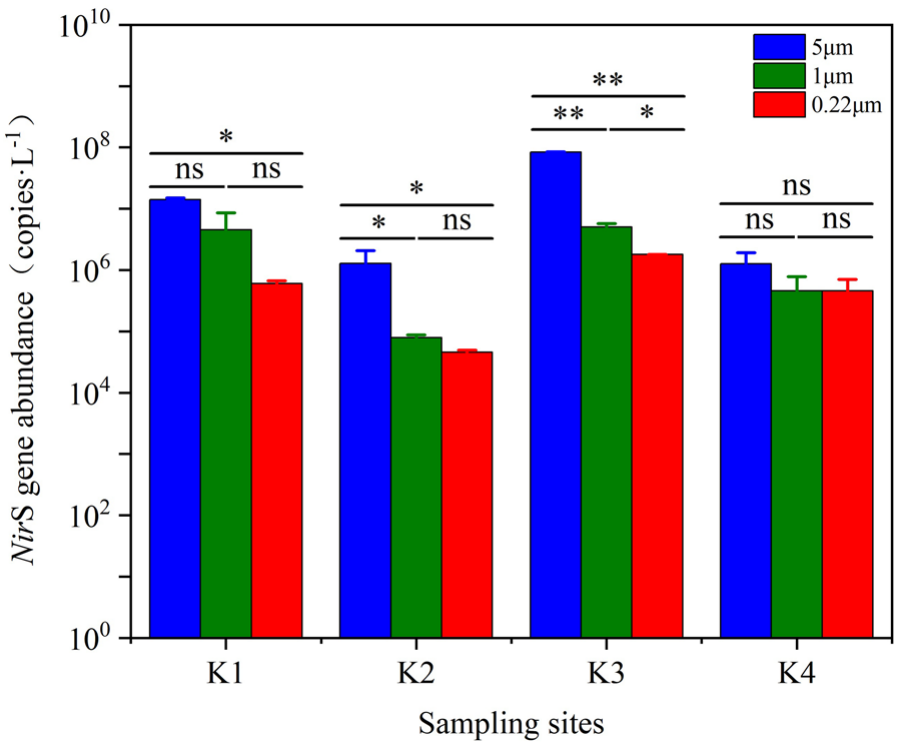
The abundance distribution of the *nir*S gene denitrifying bacteria in the high-altitude aquaculture ponds. The K1-4 in the figure is the sampling sites. The blue represents SPM with particle size of 5 μm, the green represents SPM with particle size of 1 μm, and the red represents SPM with particle size of 0.22 μm. "*, **, ns" in the figure are the results of the ANOVA (F) analysis of gene abundance on SPM with different particle sizes at each sampling site. “*” indicates a significant difference, that is, *P* < 0.05; “**” indicates an extremely significant difference, that is, *P* < 0.01; “ns” indicates no significant difference, that is, *P* > 0.05.

A standard curve was drawn based on the copy number measured using the standard sample and a correlation coefficient of R^2^ = 0.9716 was obtained. The abundance distribution of the *nir*S gene in the high-altitude aquaculture ponds was shown in Fig. 4. The abundances of the *nir*S gene ranged from 4.55 × 10^4^ to 8.41 × 10^7^ copies L^−1^ on the SPM of the high-altitude aquaculture ponds. The gene abundance ranged from 5.86 × 10^5^ to 1.41 × 10^7^ copies L^−1^ at K1 SPM with various particle sizes with orders of magnitude of 10^7^, 10^6^, and 10^5^ for 5 μm, 1 μm, and 0.22 μm SPM. The gene abundance was lowest at K2 (4.55 × 10^4^ to 1.14 × 10^6^ copies L^−1^) and highest at K3 (1.79 × 10^6^ to 8.41 × 10^7^ copies L^−1^). The gene abundance at K4 ranged from 3.98 × 10^5^ to 1.18 × 10^6^ copies L^−1^. Different from other sites, the gene abundance of 0.22 μm particle size (4.23 × 10^5^copies L^−1^) was higher than 1 μm SPM (3.98 × 10^5^ copies L^−1^).

The results of the ANOVA (F) analysis of *nir*S gene abundance on SPM with different particle sizes at each sampling site was shown in Supplementary Table S3. The abundance of *nir*S genes on SPM with different particle sizes were significantly different at K1, K2 , K3 sampling sites, which showed a trend of 5 μm > 1 μm > 0.22 μm, that is, the larger the particle size of SPM, the higher *nir*S gene abundance. SPM with a small particle size in water could provide more attachment points for denitrifying bacteria owing to its larger specific surface area^22^. The change of surface area would affect the denitrification rate and number of denitrifying bacteria^55^. However, studies also showed that denitrifying bacteria prefer to exist in the form of aggregates on the inner side of the SPM in water, and the anoxic/low oxygen micro-sites probably exist inside the SPM^22^. In this case, SPM with larger particle size could provide suitable growth environment for anaerobic denitrifying bacteria.

The abundance of functional denitrification genes between sites decreased in order of K3 > K1 > K2 > K4. This pattern may be related with the differentiation of TSS and inorganic nitrogen among the sampling sites. In this study, the TSS content in samples K3 and K1 was higher, but the inorganic nitrogen content in K3 and K1 was lower. The important components of TSS were composed of fine sediments in water and the re-suspension of the mud bottom under the action of wind and waves, whose contents could affect the number of microorganisms in water^56^. The particulate matter provides attachment sites for microorganisms and organic nitrogen, which promotes the growth of microorganisms. The TSS concentration affected the size of the SPS particles in the water, which in turn affected the number of denitrifying bacteria and the denitrification rate^55^. Suspended sediment in water promotes nitrification, denitrification, and coupled nitrification–denitrification reactions, which increase with an increase in suspended sediment content^57,58^. The sedimentation–resuspension process of the Xiaolangdi Reservoir in the Yellow River showed a significant effect on the concentration and particle size of suspended sediments^59^. Simultaneously, The sedimentation–resuspension process increases the residence time of suspended sediment in the river and promotes the growth of nitrogen cycling microorganisms, which increases the nitrogen conversion rate^60^. Inorganic nitrogen, such as NO_2_^−^-N, NH_4_^+^-N, and NO_3_^−^-N, were significantly negatively correlated with the abundance of *nir*S gene; i.e., when the content of inorganic nitrogen increased within a certain range, the abundance of *nir*S gene decreased to a certain extent^61^.

### Network analysis of *nir*S-type denitrifying bacteria

**Figure 5:**
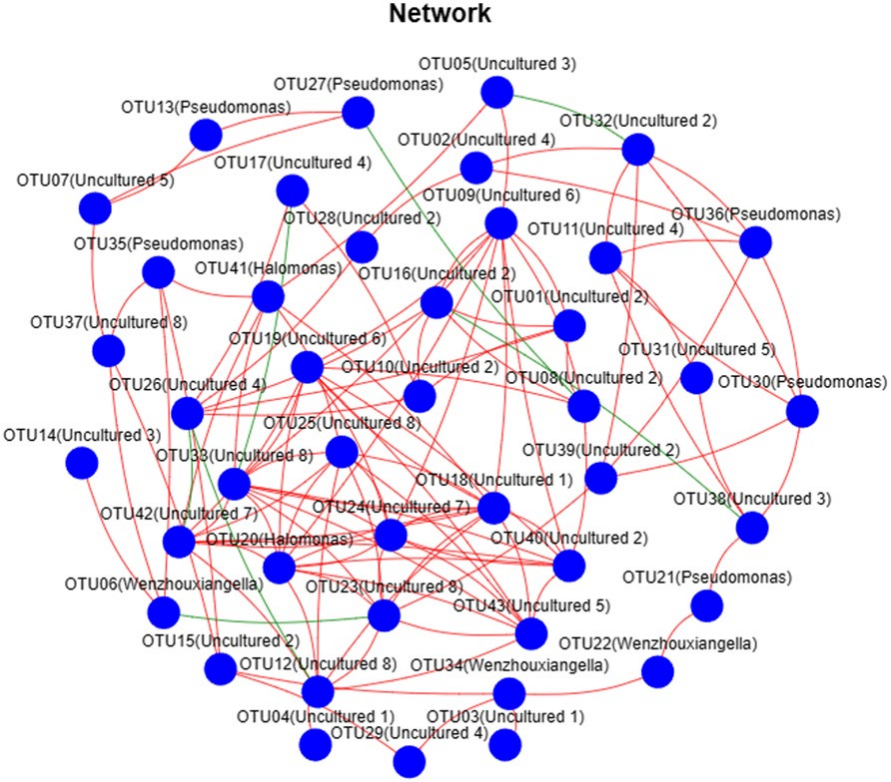
The network properties of the *nir*S-type denitrifying bacterial community at the species level in the high-altitude aquaculture ponds. The co-existence network at the species level based on the Spearman’s correlation significant analysis (*P* < 0.05). The blue circles in the diagram represent the main OTUs (species), and the connection represents the correlation between the two nodes.

Network analysis of the interaction of *nir*S-type denitrifying bacterial community based on Spearman’s correlation significance analysis was performed at the species level to explore the coexistence patterns (Fig. 5). The network analysis parameters for *nir*S-type denitrifying bacteria in the coastal high-altitude aquaculture ponds were shown in Supplementary Table S4. The number of *nir*S-type denitrifying bacteria network nodes was 43. The nodes were connected to 232 edges. The average degree and clustering coefficient was 0.44 and 5.40, respectively. The larger the clustering coefficient, the more important was the node. Clustering of OTU13 (*Pseudomonas*), OTU29 (Uncultured 4), and OTU31 (Uncultured 5) was 1, indicating the importance of these OTUs as *nir*S-type denitrifying bacteria in the high-altitude aquaculture ponds. The degrees of OTU42 (Uncultured 7), OTU18 (Uncultured 1), OTU20 (*Halomonas*), OTU24 (Uncultured 7), and OTU09 (Uncultured 6) in the network were more than ten and were highly correlated with other bacteria. A previous study demonstrated that *Pseudomonas* and *Halomonas* have crucial roles in the interaction of *nir*S-type denitrifying bacteria in coastal high-altitude aquaculture ponds, and *Pseudomonas* spp. was a key species^62^. In addition, a study of the denitrifying bacterial network in the Jinpen and Lijiahe reservoirs showed that *Paracoccus* spp. and *Staphylococcus* spp. were the key strains. This difference may be related to different water and hydrological conditions^27^.

### Effects of environmental factors on the bacterial community

**Figure 6:**
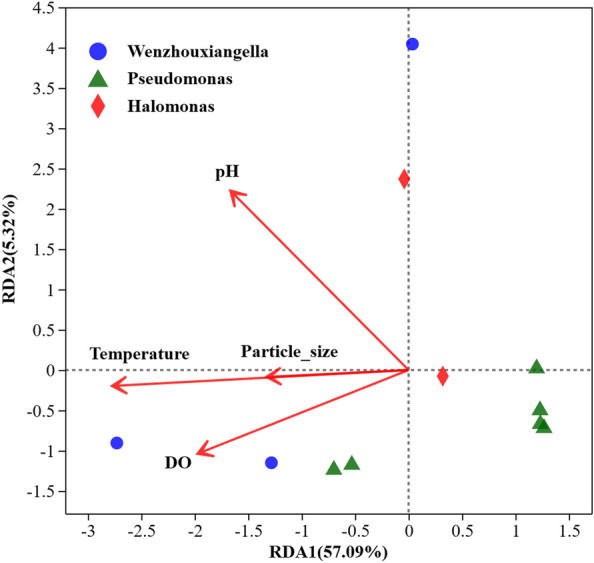
Redundancy analysis between the key genera (OTU) and environmental factors. The red arrow in the figure is an environmental parameter, and the length of the environmental factor arrow represents the degree of its influence on the bacterial community. The blue circle represents the *Wenzhouxiangella *(OTU06, OTU22, OTU34), the green triangle represents the *Pseudomonas *(OTU13, OTU21, OTU27, OTU30, OTU35, OTU36), and the red diamond represents the *Halomonas *(OTU20, OTU41).

RDA of the correlation between the community construction of denitrifying bacteria was based on the key genera (OTU) and environmental factors (Fig. 6). The explanatory power of RDA1 and RDA2 was 57.09% and 5.32%, respectively. DO, pH, temperature, and particle size were significant factors affecting changes in the community of *nir*S-type denitrifying key bacterial genera on SPM in the coastal high-altitude aquaculture pond. DO significantly affected the composition of denitrifying bacterial communities in the Jinpen and Zhoucun reservoirs^63^ but showed a weak effect on the total bacterial community structure in Hangzhou Bay^22^. These results indicated that DO could affect the community structure andgrowth metabolism^27^ of denitrifying bacteria, but it showed different effects on their community composition in various water environments.

pH is an important factor affecting the biological performance of bacteria. pH directly affects enzyme activity, which in turn affects the absorption of nutrients by microorganisms^63^. pH was identified as an important environmental factor affecting the diversity of *nir*S denitrification bacteria in this study. Similarly, pH was significantly negatively correlated with the community diversity of denitrifying bacteria in Hangzhou and Zhanjiang bays, and the interaction between pH and nitrite regulated the community diversity of denitrifying bacteria^22,61^. pH showed a key effect on the growth and metabolism of denitrifying bacteria, indicating its strong selection pressure on the growth of microorganisms and its effects on the abundance and diversity of microorganisms in the environment^30,63^. In the denitrification process, pH causes accumulation of the intermediate product, nitrite, and affects denitrification efficiency.

Zhang et al.^64^ studied the relationship between SPM and microorganisms, whose findings revealed a significant effects of particle size on the microbial community and potential denitrification capacity, and identified temperature as one of the most effective predictors of denitrification potential, similar to the results of this study. In addition, the presence of hydroxyl and amide groups on SPM affected the microbial community structure and denitrification potential^64^. When the particle size of SPM increases, the functional groups present in it also increase, which may affect the microbial community structure to a certain extent. Therefore, SPM particle size plays an important role in determining the community structure of microorganisms attached to particles.

## Conclusion

Proteobacteria were the dominant phylum of *nir*S denitrifying bacteria on the SPM of aquaculture water from the selected coastal high-altitude aquaculture ponds. Dominant genera of *nir*S-type denitrifying bacteria on SPM were *Halomonas*, *Pseudomonas*, and *Wenzhouxiangella* of Gamma-Proteobacteria. *Wenzhouxiangella* displayed the highest abundance. Network analysis revealed that *Pseudomonas* and *Halomonas* were the key genera involved in the interaction between *nir*S-type denitrifying bacteria and SPM. Moreover, the *nir*S gene abundance of denitrification exhibited a trend of 5 μm > 1 μm > 0.22 μm on SPM. DO, pH, temperature, and particle size of SPM were identified as significant factors affecting the changes in *nir*S-type denitrifying bacterial community on SPM in coastal high-altitude aquaculture ponds. These findings expand our understanding of niche differentiation and physiological characteristics of *nir*S-type denitrifying bacteria on SPM in aquaculture ecosystems. This knowledge is crucial for understanding of the microbiology of denitrification and other nitrogen cycling processes in aquaculture ecosystems, and for optimizing aquatic tailwater treatment strategies.

## Materials and methods

### Sample collection and measurements of environmental parameters

In December 2020, four sampling sites were randomly selected to collect aquaculture water samples from a high-altitude aquaculture pond at the Guanlida Marine Biological Farm located in Maoming City, Guangdong Province, China (21.55° N, 111.38° E). The aquaculture area of approximately 5.34 km^2^ is the largest marine aquaculture demonstration site and a healthy marine food production base in South China.

All water samples were collected using a 5-L gold bottle. DO, pH, and temperature of the seawater samples were measured using a Multi 3320 convenient multiparameter water quality analyzer (WTW, Munich, Germany). Turbidity of the water samples was determined using a turbidity meter. TSS were determined using the filtration constant weight method (GB11901-89). NH_4_^+^-N was determined using Nessler's reagent method (GB7879-87). NO_2_^−^–N content was determined using N-(1-naphthyl) ethylenediamine dihydrochloric acid (GB7493-87). NO_3_^−^–N content was determined using phenol disulfide (GB7480-87). TP was determined using the potassium persulfate oxidation method (GB12763.4-2007). The active phosphate levels were determined using the ascorbic acid reduction phosphomolybdate blue method (GB12763.4-2007).

SPM samples for high-throughput sequencing and qPCR analysis were filtered by a vacuum pump through 47 mm diameter 5, 1, and 0.22 μm polycarbonate membrane (Millipore, Billerica, MA, USA). The microorganisms were collected in 0.25 L water samples. The samples were named according to the combination of location and pore size (K1_5 to K4_0.22). Each filtered membrane was placed in a low temperature storage tube and stored by freezing at − 80 °C.

### DNA extraction and PCR amplification

The filter membrane containing the SPM samples was cut into pieces on an ultraclean bench using sterile scissors. DNA was extracted from each sample using the Power Water DNA Isolation Kit (MoBio, Carlsbad, CA, USA) according to the manufacturer's instructions. The quality of the DNA samples was checked using 1% agarose gel electrophoresis and NanoDrop Lite Spectrophotometer (Thermo Fisher, Waltham, MA, USA). Extracted DNA was stored at − 80 °C for subsequent molecular analysis^65^.

Denitrifying bacterial genes in different samples were amplified using PCR. Different Barcode forward primers (8 bp barcode + preprimer; Supplementary materials, Table S1) were used to distinguish the amplified DNA fragments of different samples. PCR consisted of 1 μL DNA template, 1 μL cd3aF19-30^17^, 1 μL R3cd^66^, 12.5 μL SYBR Premix Ex Taq enzyme, and 9.5 μL deionised distilled water. DNA amplification products were mixed and subjected to agarose gel electrophoresis. After determining the normal positions of the bands, DNA amplification products were purified and collected using a gel recovery kit (TaKaRa Bio, Dalian, China).

### High-throughput sequencing analysis

The purified products were sequenced on the Illumina HiSeq platform (Genewiz Corporation, Suzhou, China) and analyzed using mothur (version 1.9.5) according to the high-throughput sequencing standard procedure reported by Sun et al.^65^. Microbial community structure and diversity were analyzed using the Mothur platform and standard operating procedures. Effective raw data were obtained by removing barcodes, noise reduction, and pruning the sample sequence. Sequences with lengths less than 380 bp were removed. The final sequence obtained by preprocessing was the main OTU sequence after removing rare OTUs (sequence number < 0.2%). A total of 46 OTU sequences accounted for 91.7% of the total sequence number. According to the alpha diversity analysis of the files generated by Mothur^65^, alpha diversity was related to richness and diversity^65^. The Chao1 and ACE richness indices and Shannon and Simpson diversity indices were determined. The Shannon-Winner curve was drawn using Origin software (OriginLab Corporation, Northampton, MA, USA) to illustrate the feasibility of high-throughput sequencing data. A Rank-Abundance curve was drawn to illustrate the species richness and evenness of sample diversity. Beta diversity analysis was performed on files generated by Mothur, and PCoA and NMDS were performed using SigmaPlot 12.0^65^. To measure the differences in species composition between the different regions, the diversity between the communities was compared and analysed. Reference sequences of the *nir*S were derived from Fungene and NCBI (https://www.ncbi.nlm.nih.gov/), as previously described^25^. A neighbour-joining phylogenetic tree was constructed and visualised using MEGA7.0 (https://www.megasoftware.net/) and EvolView^67^. A heat map was plotted using Microsoft Excel (Microsoft Excel 2016). Based on the abundance distribution of the main OTUs in each sample and the sample clustering results, Origin software and R language were used to draw the distribution and heat map of the denitrifying bacterial community structure.

### qPCR analysis of *nir*S functional genes for denitrification

The PCR product of the target gene was ligated to the pMD19-T Simple Vector (TaKaRa Bio), and the DNA Extraction Kit Ver.4.0 kit (TaKaRa Bio) was used for ligation and transformation^65^. A linearised plasmid containing the cloned bacterial *nir*S gene was continuously diluted ten times (10^10^–10^3^) to obtain a standard curve. Three samples and standard reactions were performed using a CFX 96C 1000TM thermal cycler (Bio-Rad, Hercules, CA, USA), and the average values were calculated. The system consisted of 1 μL DNA template, 0.2 μL cd3aF^17^ (5′-GTSAACGTSAAGGARACSG-3′), 0.2 μL R3cd^66^ (5′-GASTTCGGRTGSGTCTTGA-3′), 7.5 μL SYBR Premix Ex Taq enzyme (Promega), 0.3 μL ROX (Promega, America) and 5.8 μL deionised distilled water. PCR reaction conditions were pre-denaturation at 95 °C for 10 min, and 40 cycles of denaturation at 95 °C for 5 s, annealing at 55 °C for 30 s, extension at 72 °C for 30 s. The specificity of amplification was verified by observing the melting curve. The PCR amplification efficiency was 83–100.7%, and the correlation coefficient (R^2^) was greater than 0.99. Finally, the gene copy number was calculated using regression analysis.

### Statistical analyses

SPSS Statistics 26 (IBM, Armonk, NY, USA) was used to analyse the differences in environmental factors at each sampling site and differences in *nir*S gene abundance on SPM with different particle sizes within each sampling site. *P*-values less than 0.05 indicated, a significant difference between the sites of environmental factors, and values less than 0.01 indicated very significant difference. The correlation between the diversity index of the bacterial community and environmental factors in the high-altitude aquaculture ponds was plotted using the R language corrplot function^65^. Spearman’s correlation analysis was performed using SPSS software at the species level. Data with robust (|r|> 0.5) and significant (*P* < 0.05) associations were selected to construct the network model^27^. Canoco5 software was used to analyse the gene abundance and environmental factors in the water samples^65^. The main environmental factors affecting the key genera (OTU) of community structure were analyzed by RDA^65^.

### Nucleotide sequence accession numbers

The datasets are found in the National Genomics Data Center (NGDC) and part of the China National Center for Bioinformation (CNCB) [accession number CRA010569].

### Supplementary Information

Supplementary Information.

## Data Availability

The datasets presented in this study can be found in online repositories. The names of the repository/repositories and accession number(s) can be found below: National Genomics Data Center (NGDC), part of the China National Center for Bioinformation (CNCB) [accession: CRA010569].
