# Supplementary material for: Diversity, community structure, and abundance of nirS-type denitrifying bacteria on suspended particulate matter in coastal high-altitude aquaculture pond water
Source: Sci Rep. 2024 Mar 7;14:5594. doi: 10.1038/s41598-024-56196-x (PMC10920899; doi:10.1038/s41598-024-56196-x)
Supplement: Supplementary file 1 — Supplementary Information. [file 41598_2024_56196_MOESM1_ESM.docx]

Supplementary material

Supplementary Table S1. Primers and reaction procedures used for PCR amplification.

| Sample | Primer | Barcod | reaction system | reaction condition |
| --- | --- | --- | --- | --- |
| K1_5 | cd3aF19 | ACCATGCA | Total system 25μL  ( template 1 μL ;  enzyme 12.5 μL ;  positive primers  cd3aF 1 μL ;  reverse primers  R3cd 1 μL ;  ddH2O 9.5μL ) | Pre-denaturation at 95 °C for 3 min ;  95 °C denaturation 45S ;  annealing at 55 °C for 45 s ;  72 °C extension for 40 s ;  a total of 35 cycles |
| K1_1 | cd3aF20 | ACCATGGT |  |  |
| K1_0.22 | cd3aF21 | ACCTACCT |  |  |
| K2_5 | cd3aF22 | ACCTACGA |  |  |
| K2_1 | cd3aF23 | ACCTAGCA |  |  |
| K2_0.22 | cd3aF24 | ACCTAGGT |  |  |
| K3_5 | cd3aF25 | ACCTCAAG |  |  |
| K3_1 | cd3aF26 | ACCTCATC |  |  |
| K3_0.22 | cd3aF27 | ACCTCTAC |  |  |
| K4_5 | cd3aF28 | ACCTCTTG |  |  |
| K4_1 | cd3aF29 | ACCTGAAC |  |  |
| K4_0.22 | cd3aF30 | ACCTGATG |  |  |

NOTE: The K1-4 is the sampling sites. The number 5, 1, and 0.22 is particle sizes of 5, 1, and 0.22 μm on SPM.

Supplementary Table S2. ANOVA (F) of environmental parameters between different sampling sites.

| Site/Site | K1/K2 | K1/K3 | K1/K4 | K2/K3 | K2/K4 | K3/K4 |
| --- | --- | --- | --- | --- | --- | --- |
| F | 4.850 | 0.182 | 4.183 | 9.537 | 0.376 | 7.889 |
| Sig. | 0.040* | 0.675 | 0.054 | 0.006** | 0.546 | 0.011* |

NOTE: The K1-4 is the sampling sites. “*” in the table indicates a significant difference, that is, *P < 0.05*; “**” indicates that the difference is extremely significant, that is, *P < 0.01.*


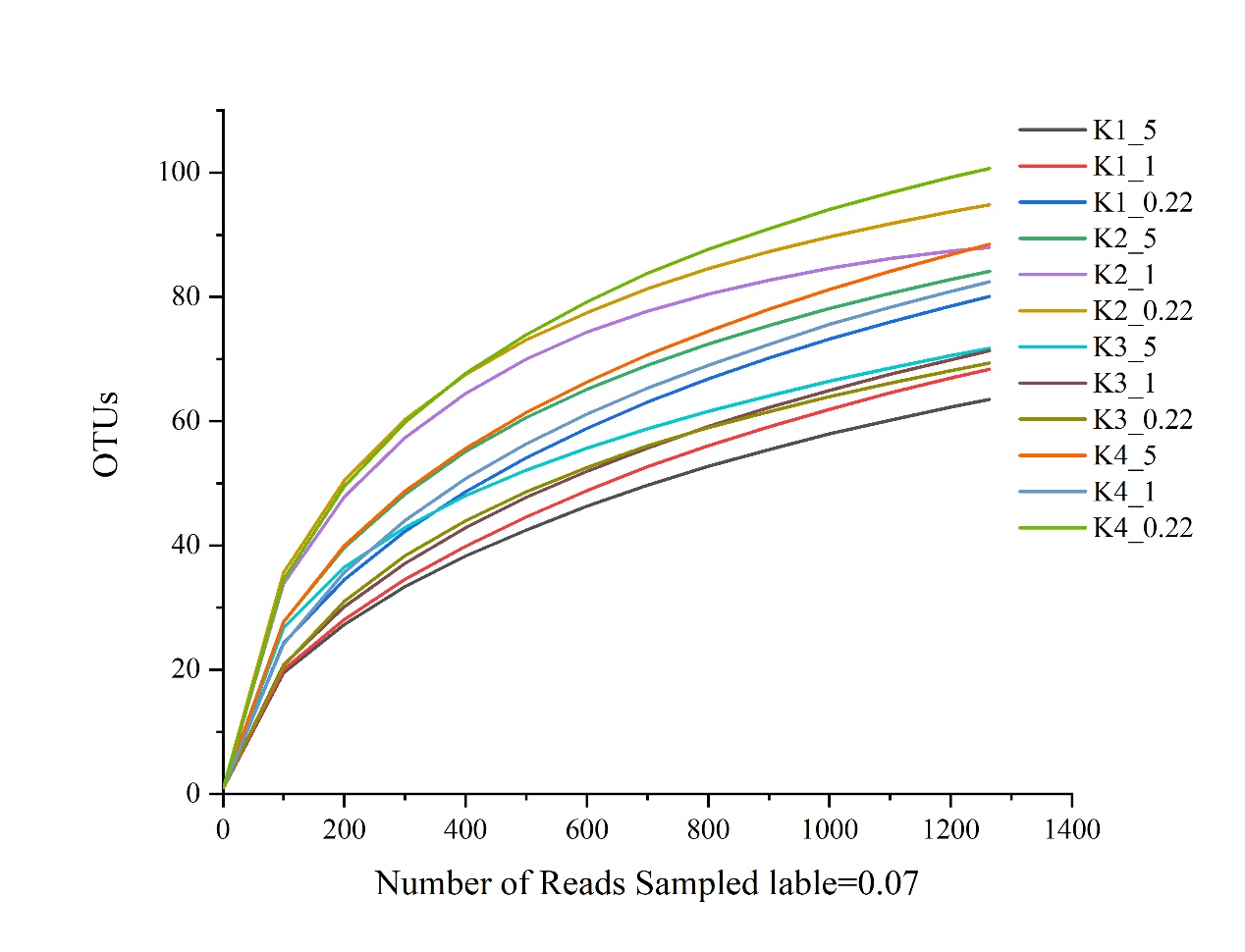


Supplementary Figure S1. The dilution curve diagram. The flattening indicates that the sequencing depth basically covers all species in all samples, and the species diversity in the samples has been basically detected.


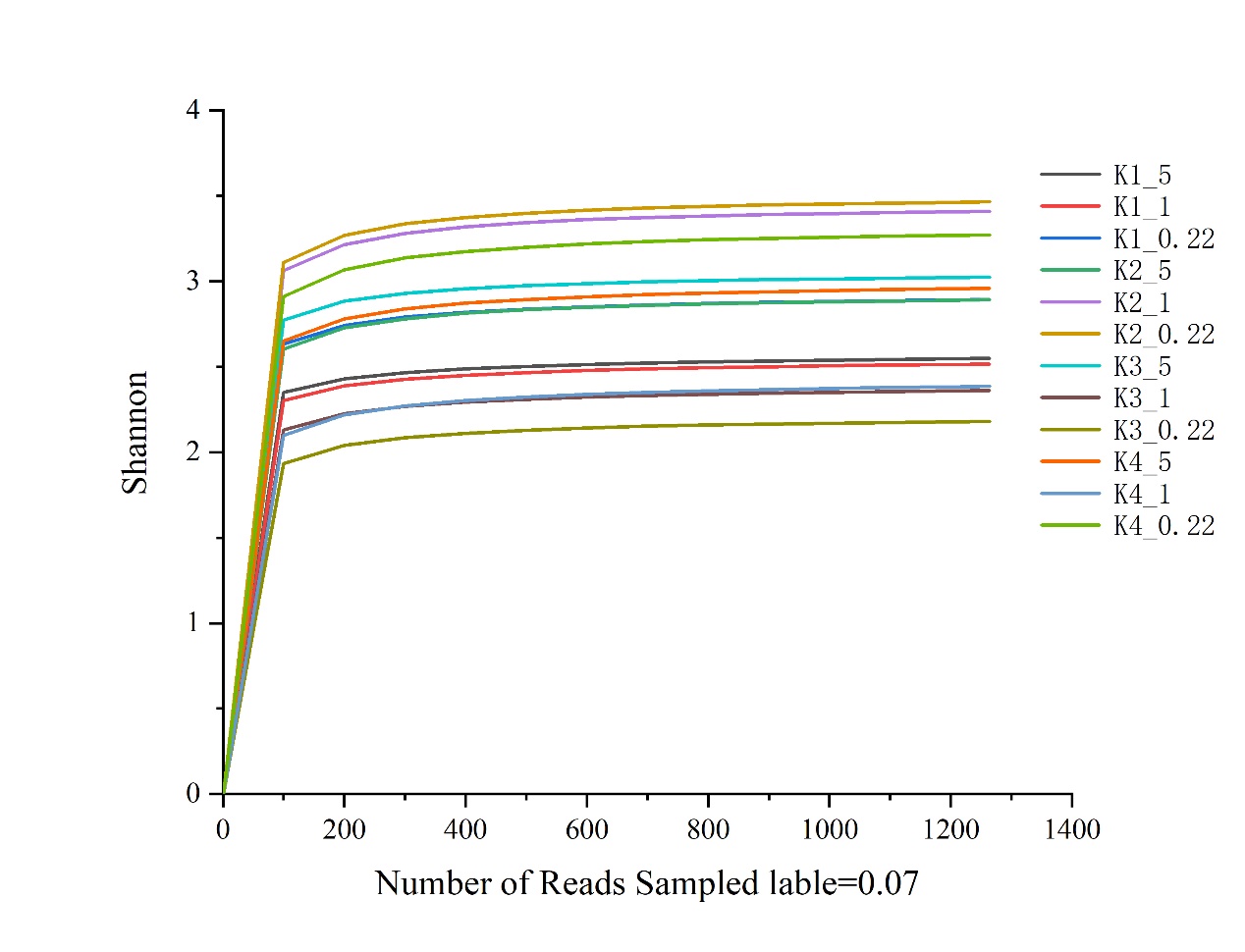


Supplementary Figure S2. The Shannon index diagram. The gentle trend indicated that the amount of data sequenced in this study was large enough to reflect the vast majority of microbial information in all samples.


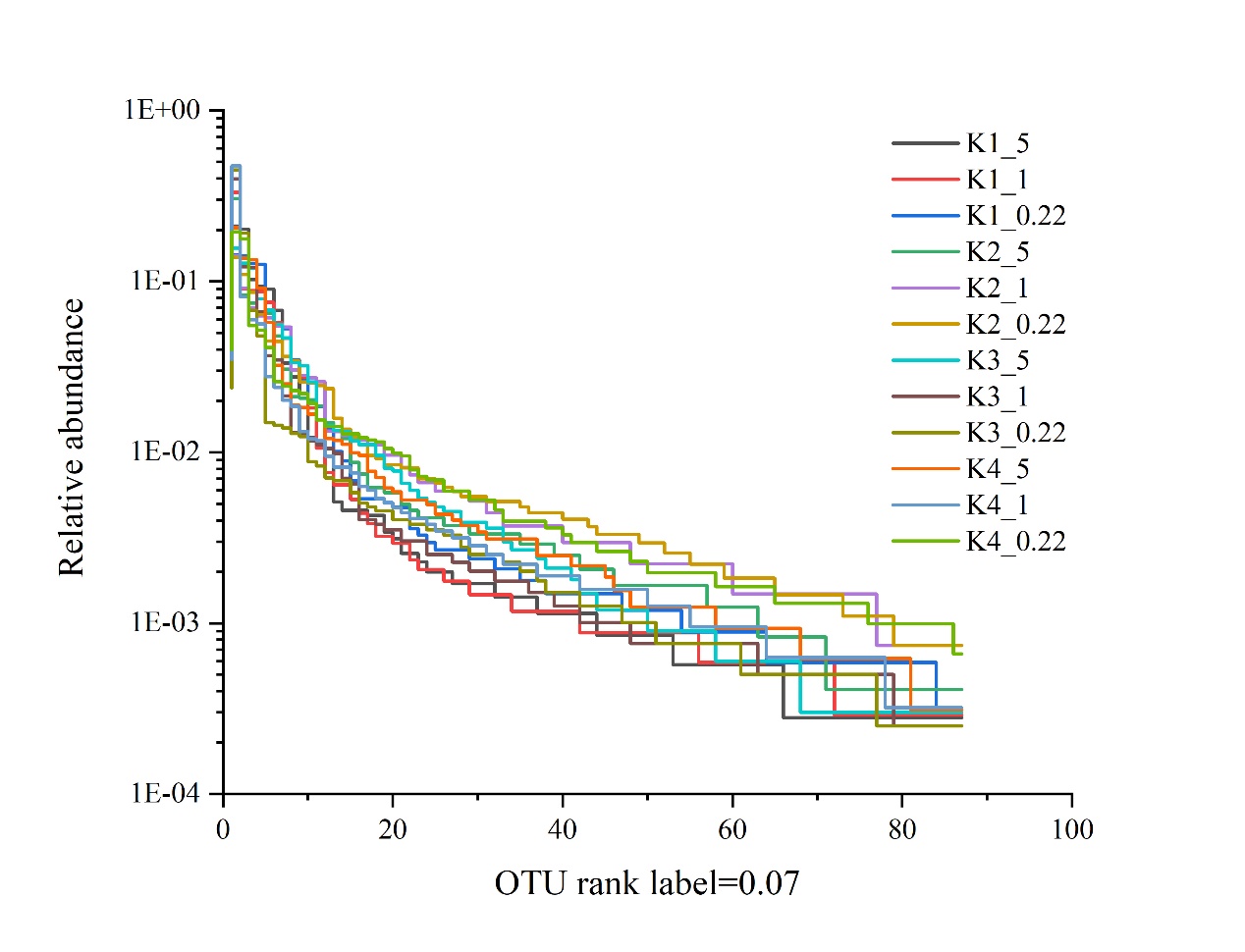


Supplementary Figure S3. The Rank-Abundance curve diagram. The Rank-Abundance graph. The rapid decline indicated that the proportion of dominant bacteria in all samples was high and the diversity was low.

Supplementary Table S3. ANOVA (F) of gene abundance on SPM with

different particle sizes at each sampling site.

| sampling site | particle size | Sig. | abstract |
| --- | --- | --- | --- |
| SPM in K1 | 5 vs. 1 | 0.054 | ns |
|  | 5 vs. 0.22 | 0.023 | * |
|  | 1 vs. 0.22 | 0.350 | ns |
| SPM in K2 | 5 vs. 1 | 0.016 | * |
|  | 5 vs. 0.22 | 0.015 | * |
|  | 1 vs. 0.22 | 0.989 | ns |
| SPM in K3 | 5 vs. 1 | <0.001 | ** |
|  | 5 vs. 0.22 | <0.001 | ** |
|  | 1 vs. 0.22 | 0.0245 | * |
| SPM in K4 | 5 vs. 1 | 0.306 | ns |
|  | 5 vs. 0.22 | 0.304 | ns |
|  | 1 vs. 0.22 | >0.999 | ns |
| SPM in all sampling site | all | <0.001 | ** |

NOTE: The K1-4 is the sampling sites. "*, **, ns" in the table are the results of the ANOVA (F) analysis of gene abundance on SPM with different particle sizes at each sampling site. “*” indicates a significant difference, that is, *P<0.05*; “**” indicates an extremely significant difference, that is, *P<0.01*；”ns” indicates no significant difference，that is, *P>0.05.*

Supplementary Table S4. Network analysis parameters of *nir*S-type denitrifying bacteria in coastal high-altitude aquaculture ponds.

| Node Name | degree | clustering | Degree  Centrality | Closeness  Centrality | Betweenness  Centrality |
| --- | --- | --- | --- | --- | --- |
| OTU41(*Halomonas*) | 6 | 0.40 | 0.14 | 0.39 | 0.13 |
| OTU42(Uncultured 7) | 10 | 0.58 | 0.24 | 0.39 | 0.05 |
| OTU25(Uncultured 8) | 9 | 0.75 | 0.21 | 0.37 | 0.01 |
| OTU18(Uncultured 1) | 12 | 0.65 | 0.29 | 0.40 | 0.04 |
| OTU33(Uncultured 8) | 12 | 0.58 | 0.29 | 0.40 | 0.06 |
| OTU05(Uncultured 3) | 3 | 0.00 | 0.07 | 0.37 | 0.06 |
| OTU35(*Pseudomonas*) | 4 | 0.33 | 0.10 | 0.34 | 0.12 |
| OTU19(Uncultured 6) | 9 | 0.75 | 0.21 | 0.36 | 0.02 |
| OTU40(Uncultured 2) | 9 | 0.75 | 0.21 | 0.36 | 0.02 |
| OTU20(*Halomonas*) | 11 | 0.73 | 0.26 | 0.38 | 0.02 |
| OTU24(Uncultured 7) | 10 | 0.78 | 0.24 | 0.38 | 0.01 |
| OTU12(Uncultured 8) | 8 | 0.50 | 0.19 | 0.37 | 0.07 |
| OTU26(Uncultured 4) | 7 | 0.24 | 0.17 | 0.38 | 0.10 |
| OTU43(Uncultured 5) | 9 | 0.78 | 0.21 | 0.35 | 0.01 |
| OTU23(Uncultured 8) | 9 | 0.53 | 0.21 | 0.40 | 0.17 |
| OTU09(Uncultured 6) | 10 | 0.44 | 0.24 | 0.40 | 0.11 |
| OTU08(Uncultured 2) | 6 | 0.40 | 0.14 | 0.35 | 0.10 |
| OTU17(Uncultured 4) | 3 | 0.33 | 0.07 | 0.33 | 0.01 |
| OTU04(Uncultured 1) | 1 | 0.00 | 0.02 | 0.27 | 0.00 |
| OTU39(Uncultured 2) | 4 | 0.50 | 0.10 | 0.34 | 0.08 |
| OTU06(*Wenzhouxiangella*) | 4 | 0.17 | 0.10 | 0.35 | 0.10 |
| OTU01(Uncultured 2) | 5 | 0.60 | 0.12 | 0.35 | 0.01 |
| OTU16(Uncultured 2) | 6 | 0.40 | 0.14 | 0.38 | 0.14 |
| OTU10(Uncultured 2) | 4 | 0.67 | 0.10 | 0.32 | 0.01 |
| OTU38(Uncultured 3) | 5 | 0.20 | 0.12 | 0.34 | 0.16 |
| OTU27(*Pseudomonas*) | 3 | 0.33 | 0.07 | 0.30 | 0.06 |
| OTU03(Uncultured 1) | 1 | 0.00 | 0.02 | 0.20 | 0.00 |
| OTU34(*Wenzhouxiangella*) | 4 | 0.17 | 0.10 | 0.25 | 0.08 |
| OTU32(Uncultured 2) | 6 | 0.40 | 0.14 | 0.33 | 0.06 |
| OTU02(Uncultured 4) | 3 | 0.33 | 0.07 | 0.27 | 0.01 |
| OTU28(Uncultured 2) | 2 | 0.00 | 0.05 | 0.30 | 0.02 |
| OTU36(*Pseudomonas*) | 5 | 0.60 | 0.12 | 0.30 | 0.02 |
| OTU30(*Pseudomonas*) | 5 | 0.60 | 0.12 | 0.32 | 0.03 |
| OTU11(Uncultured 4) | 5 | 0.50 | 0.12 | 0.30 | 0.03 |
| OTU21(*Pseudomonas*) | 2 | 0.00 | 0.05 | 0.28 | 0.07 |
| OTU22(*Wenzhouxiangella*) | 2 | 0.00 | 0.05 | 0.25 | 0.05 |
| OTU31(Uncultured 5) | 2 | 1.00 | 0.05 | 0.27 | 0.00 |
| OTU14(Uncultured 3) | 1 | 0.00 | 0.02 | 0.26 | 0.00 |
| OTU37(Uncultured 8) | 4 | 0.33 | 0.10 | 0.31 | 0.07 |
| OTU15(Uncultured 2) | 4 | 0.33 | 0.10 | 0.29 | 0.11 |
| OTU29(Uncultured 4) | 2 | 1.00 | 0.05 | 0.24 | 0.00 |
| OTU07(Uncultured 5) | 3 | 0.33 | 0.07 | 0.28 | 0.03 |
| OTU13(*Pseudomonas*) | 2 | 1.00 | 0.05 | 0.25 | 0.00 |
